# Determinants associated with receiving a medical appointment through the primary care access points for unattached adults in Quebec: A cross-sectional study

**DOI:** 10.1177/08404704241301773

**Published:** 2024-11-24

**Authors:** Mylaine Breton, Catherine Lamoureux-Lamarche, Véronique Deslauriers, Djamal Berbiche, Maude Laberge, Mélanie Ann Smithman, Annie Talbot, Isabelle Gaboury, Marie-Pascale Pomey, Marie Beauséjour

**Affiliations:** 1 7321 Université de Sherbrooke, Longueuil, Québec, Canada.; 2 12369 Université Laval, Québec City, Québec, Canada.; 3 University of Toronto, Toronto, Ontario, Canada.; 4 5622 Université de Montréal, Montréal, Québec, Canada.

## Abstract

Canada is experiencing an unprecedented primary care crisis, with 6.5 million Canadians reporting lacking a family physician, including 31% of the Quebec population. To address this problem, the province of Quebec implemented primary care access points (in French, they are *Guichets d’accès à la première ligne*, or GAPs) to help unattached patients navigate and access primary care services while awaiting attachment. We aimed to examine the determinants associated with unattached patients receiving a medical appointment compared to another service through the GAP. Cross-sectional data (n = 13,291) from two GAPs were collected (June 2022 to March 2023). Multivariable logistic regression was carried out. Being younger, calling for an acute health problem, medication renewal or to have administrative documentation filled, having a physical or mental health problem, and using GAP A (compared to GAP B) were associated with an increased likelihood of receiving a medical appointment. This study is the first to document the characteristics of patients using the GAP and their needs.

## Introduction

Canada is experiencing an unprecedented primary care crisis with access challenges at the centre.^1,2^ An estimated 6.5 million Canadians do not have a regular Primary Care Provider (PCP), with the province of Quebec having the highest rate of unattachment (31%).^3,4^ Attachment to a PCP and access to care are key components of high-performing primary care system,^5-7^ contributing to better health outcomes, continuity and coordination of care, and lower emergency department use and healthcare costs.^8-14^ Although access issues in Quebec are not new, they are currently exacerbated by the primary healthcare labour shortage, including increasing numbers of family physicians retiring, vacant family medicine residency positions, and growing numbers of unattached patients.^15-17^

To address this access challenge, over the past 15 years, 8 Canadian provinces, including Quebec, implemented centralized waiting lists (CWLs) for unattached patients.^18,19^ These CWLs are designed to centralize all requests for attachment and match patients with a PCP based on territory, priority criteria and PCP availability.^18^ As of May 2024, 1.55 million Quebecers (17% of the population) were registered on the CWL awaiting attachment.^20,21^

Although the CWL in Quebec has led to the attachment of over a million patients since its implementation, the number of unattached patients and the waiting time to be attached have continued to rise in the last few years.^22,23^ The Quebec Ministry of Health mandated, in 2022, province-wide implementation of primary care access points (*Guichets d’accès à la première ligne*; GAP) for unattached patients and incentivized family physicians to offer medical appointments.

**Figure 1.**
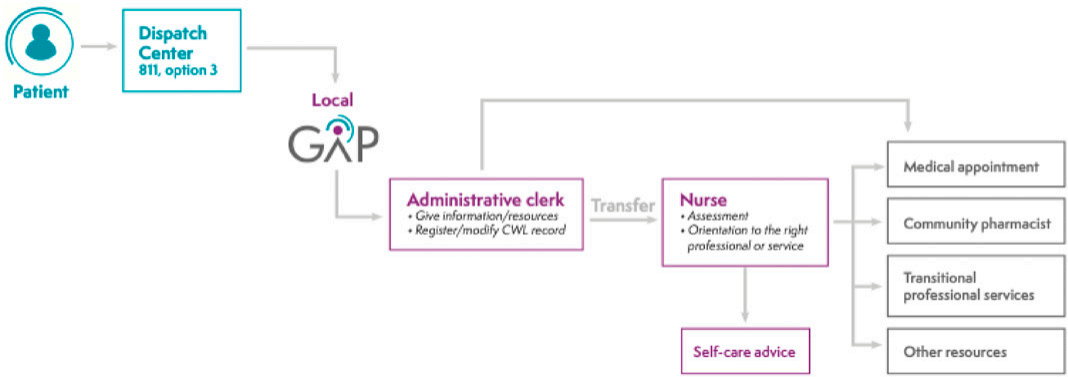
Primary Care Access Point structure.

The GAP is an organizational innovation, implemented within regions, aiming to help unattached patients navigate and access primary care services while awaiting attachment. It operates on a geographical basis and is linked to the local CWL. Figure 1 details GAP structure and functioning. First, unattached patients call the GAP dispatch centre. If the patient is eligible (i.e., already registered on the CWL), the call is transferred to their local GAP, where an administrative clerk gives information/resources and registers/modifies the patient’s CWL record, if needed. Then, if the patient needs a medical appointment or to be assessed by a nurse, the most appropriate of the following decisions is made based on a decision tree: (1) give a medical appointment without transferring the patient to a registered nurse, (2) transfer the patient to a GAP registered nurse, or (3) add the patient to a waiting list to be called by a GAP nurse. The patient is oriented to the most relevant professional or service according to their need and available professional resources, including a medical appointment with a family physician, referral to a community pharmacist, referral to GAP transitional professional services for patients with unstable chronic diseases, and advice to consult a community resource. This ensures better allocation of scarce medical appointments for patients requiring a family physician. Although the innovation was first piloted in a small region,^24^ data on its effects and the population using the service are scarce.

Our research team previously examined characteristics of 1,323 unattached paediatric patients using the GAP and associations with the need for a medical appointment.^25^ We found that being 0-5 years old, being first assessed by a nurse (Info-Health), and calling for medication renewal were associated with an increased likelihood of needing a medical appointment.^25^ Although this study was the first to document the determinants of needing a medical appointment in unattached GAP patients compared to another service through the GAP, it was limited to the paediatric population (aged <18 years), which has different characteristics and needs than the adult population. Therefore, we aim to examine, in unattached adults, the socio-demographic, economic, and clinical characteristics associated with receiving a medical appointment compared to another service through the GAP. Our study will enable a better understanding of who uses GAP services in order to improve access in case of clinical needs.

## Method

This retrospective study was conducted in a region with two local health territories and two GAPs (one per local health territory) with over 78,000 eligible unattached patients. Cross-sectional data were extracted from GAP Electronic Medical Records (EMRs), which includes data collected during the call and from CWL records.

The study sample included all patients aged over 18 years who called the GAP between June 2022 and March 2023. Given that nearly 70% of GAP patients called only once during the study period, and to facilitate data management and analysis, only one call per patient was included in the study. For patients with more than one call, the following strategy was used to identify the included call. For patients who received a medical appointment from the administrative clerk or nurse, the first call during the study period with a medical appointment was considered. For patients who never received a medical appointment, the first call assessed by a registered nurse was prioritized. If no calls were assessed by a registered nurse, the first call answered by the administrative clerk was considered. The study sample was n = 13,291.

### Measures

#### Receiving a medical appointment

Receiving a medical appointment through the GAP was dichotomized (yes/no). Medical appointments are face-to-face consultations with a family physician in a primary care clinic located on the local health territory. Patients who did not have a medical appointment may have received other services or references from the GAP, including self-care advice, general information and referral to GAP transitional professional services, a community pharmacist, another health professional, or a community resource.

#### Independent variables

Socio-demographic and economic characteristics included age, sex, and social and material area-deprivation indexes.^26,27^ Clinical and health characteristics included priority code for attachment to a family physician, having a chronic disease, main reason for calling and type of health problem. The GAP was categorized as GAP A or GAP B depending on the patient’s address. Each variable’s definition and operationalization are detailed in Table 1.

**Table 1.** Independent variables definition and operationalization.

Variables	Definition/categories
Socio-demographic and economic characteristics	
Age	18-45/46-65/66 years and older
Sex	Female/Male
Social and material area-deprivation indexes	These area-based indexes are developed using Canadian census data and assigned based on the patient’s postal code.^26,27^ The social deprivation index is based on the proportion of residents aged 15 years and older who are (1) from single-parent families; (2) living alone; and (3) divorced, separated, or widowed. The material deprivation index relies on the proportion of residents aged 15 years or older who are (1) employed, (2) without a high school diploma, and (3) their average individual income. They range between 1 (most privileged) and 5 (least privileged)^26,27^
Clinical and health characteristics	
Priority code for attachment to a family physician	The priority code is an indicator developed by the Quebec Ministry of Health and is determined by a nurse working at the CWL.^28^ The codes range from A to E, with A being the most urgent (within 1 week) and E a regular registration (within 3 months). Codes are assigned according to self-declared information, including age, chronic conditions, and hospitalizations in the last month
Having a chronic disease	Chronic diseases are indicated by the presence of at least one health vulnerability code defined by Quebec’s public health insurance plan and attributed to specific physical and mental health conditions^29^
The main reason for the call	The main reason for the call was determined by the administrative clerk or nurse and categorized as follows: (1) acute health problem, (2) medication renewal, (3) administrative health documentation to be filled, and (4) other (e.g., general information, registration to the CWL, and follow-up)
Type of health problem	The type of health problem was determined by the nurse based on the main health problem discussed during the call and included a list of 16 health systems/categories. For analysis purposes, these categories were later merged as follows: (1) no health problem, (2) physical health, (3) mental health, and (4) other health problems. Given that this variable was not collected by the administrative clerk, information on the main reason for the call was explored more closely. Medication renewals, general information, registration to the CWL, and forms needing to be filled (n = 918), which represented 62% of all calls answered by the administrative clerk, were categorized as “no health problem”
GAP characteristic	
GAP localization	A/B

### Analysis

Descriptive analysis was conducted to describe the study sample. Bivariate and multivariable binomial logistic regressions were used to assess the socio-demographic, economic, and clinical factors as well as the GAP-related characteristic associated with receiving a medical appointment. Adjusted Odds Ratios (AORs) are presented with 95% Confidence Intervals (CIs). Statistical analyses were carried out using SPSS V29.0 and SAS V9.4.

## Results

The study sample was mostly female (58.4%) with a mean age of 53.7 years. Over 50% of the sample was considered vulnerable based on their priority code (codes A, B, and C), and 32% had at least one chronic disease. The main reason for calling was an acute health problem (71%), whereas physical and mental health problems represented 53% and 7% of cases, respectively. GAP A received 55% of requests. A total of 64% of the sample received a medical appointment.

Table 2 presents bivariate logistic regression models. Age, social deprivation index, priority code for attachment to a family physician, main reason for calling, type of health problem, and GAP localization were associated with receiving a medical appointment.

**Table 2.** Characteristics of the study sample and factors associated with receiving a medical appointment through the GAP.

	Total sample N = 13,291	Receiving a medical appointment N = 8,540 (64%)	Not receiving a medical appointment N = 4,751 (36%)	Odds ratio (95% CI)	Adjusted odds ratio (95% CI) N = 12,092
Socio-demographic and economic characteristics					
Sex					
Female	7,767 (58.4)	4,979 (58.3)	2,788 (58.7)	0.984 (0.916 - 1.058)	0.991 (0.912 - 1.078)
Male	5,524 (41.6)	3,561 (41.7)	1,963 (41.3)	Reference	Reference
Missing	0				
Age					
18-45 years	4,737 (35.6)	3,164 (37.1)	1,573 (33.1)	1.389 (1.273 - 1.515)	1.201 (1.064 - 1.355)
46-65 years	4,507 (33.9)	2,982 (34.9)	1,525 (32.1)	1.350 (1.236 - 1.474)	1.244 (1.114 - 1.389)
66 years and over	4,047 (30.4)	2,394 (28.0)	1,653 (34.8)	Reference	Reference
Missing	0				
Social deprivation index					
1 (most privileged)	3,283 (24.7)	2,099 (24.6)	1,184 (24.9)	1.043 (0.941 - 1.156)	0.972 (0.860 - 1.099)
2	2,016 (15.2)	1,288 (15.1)	728 (15.3)	1.041 (0.926 - 1.170)	1.056 (0.918 - 1.215)
3	2,572 (19.4)	1,669 (19.5)	903 (19.0)	1.087 (0.974 - 1.213)	1.073 (0.939 - 1.226)
4	2,414 (18.2)	1,593 (18.7)	821 (17.3)	1.141 (1.020 - 1.277)	1.089 (0.952 - 1.245)
5 (least privileged)	2,997 (22.5)	1,887 (22.1)	1,110 (23.4)	Reference	Reference
Missing	9 (0.1)	4 (0.05)	5 (0.1)		
Material deprivation index					
1 (most privileged)	3,025 (22.8)	1,892 (22.2)	1,133 (23.9)	0.916 (0.811 - 1.034)	0.907 (0.783 - 1.051)
2	2,904 (21.8)	1,887 (22.1)	1,017 (21.4)	1.017 (0.899 - 1.151)	1.005 (0.865 - 1.167)
3	2,821 (21.2)	1,803 (21.1)	1,018 (21.4)	0.971 (0.858 - 1.099)	0.937 (0.808 - 1.086)
4	2,753 (20.7)	1,805 (21.1)	948 (20.0)	1.044 (0.921 - 1.183)	0.964 (0.831 - 1.118)
5 (least privileged)	1,779 (13.4)	1,149 (13.5)	630 (13.2)	Reference	Reference
Missing	9 (0.1)	4 (0.05)	5 (0.1)		
Clinical and health characteristics					
Priority code for attachment to a family physician					
A (most urgent)	376 (2.8)	233 (2.7)	143 (3.0)	0.844 (0.675 - 1.055)	0.801 (0.610 - 1.051)
B	901 (6.8)	554 (6.5)	347 (7.3)	0.827 (0.707 - 0.967)	1.055 (0.858 - 1.298)
C	5,739 (43.2)	3,664 (42.9)	2,075 (43.7)	0.914 (0.830 - 1.007)	1.123 (0.975 - 1.295)
D	2,990 (22.5)	2,001 (23.4)	989 (20.8)	1.047 (0.937 - 1.171)	1.085 (0.956 - 1.231)
E (least urgent)	2,615 (19.7)	1,723 (20.2)	892 (18.8)	Reference	Reference
Missing	670 (5.0)	365 (4.3)	305 (6.4)		
Having a chronic disease					
Yes	4,257 (32.0)	2,726 (31.9)	1,531 (32.2)	0.986 (0.914 - 1.064)	0.980 (0.878 - 1.096)
No	9,034 (68.0)	5,814 (68.1)	3,220 (67.8)	Reference	Reference
Missing	0				
Main reason for call					
Acute health problem	9,465 (71.2)	6,680 (78.2)	2,785 (58.6)	10.094 (8.774 - 11.612)	5.707 (4.835 - 6.736)
Medication renewal	2,078 (15.6)	1,360 (15.9)	718 (15.1)	7.971 (6.787 - 9.361)	7.932 (6.681 - 9.418)
Administrative health documentation to fill	347 (2.6)	231 (2.7)	116 (2.5)	8.380 (6.464 - 10.865)	10.070 (7.614 - 13.318)
Other (general information, resources, and CWL)	1,401 (10.6)	269 (3.2)	1,132 (23.8)	Reference	Reference
Missing	0				
Type of health problem					
No health problem	2,789 (21.0)	1,172 (13.7)	1,617 (34.0)	0.441 (0.392 - 0.497)	0.578 (0.497 - 0.673)
Physical health	7,095 (53.4)	5,265 (61.7)	1,830 (38.5)	1.752 (1.576 - 1.947)	1.651 (1.472 - 1.852)
Mental health	879 (6.6)	705 (8.3)	174 (3.7)	2.467 (2.041 - 2.981)	2.461 (1.998 - 3.030)
Other health problem	1,958 (14.7)	1,217 (14.2)	741 (15.6)	Reference	Reference
Missing	570 (4.3)	181 (2.1)	389 (8.2)		
GAP characteristic					
GAP localization					
A	7,289 (54.8)	4,951 (58.0)	2,338 (49.2)	1.424 (1.326 - 1.529)	1.428 (1.311 - 1.555)
B	6,002 (45.2)	3,589 (42.0)	2,413 (50.8)	Reference	Reference
Missing	0				

CI: confidence interval.

GAP: Primary Care Access Point.

Significant results (*P* < 0.05) are in bold.

Table 2 presents multivariable logistic regression models. Younger patients (≤65 years) were more likely to receive a medical appointment than older adults. Patients calling for acute health problems (AOR: 5.707, 95% CI: 4.835-6.736), medication renewals (AOR: 7.932, 95% CI: 6.681-9.418), or to have administrative health documentation filled (AOR: 10.070, 95% CI: 7.614-13.318) were more likely to receive an appointment than patients calling for another reason. Compared to other health problems, patients with a physical (AOR: 1.651, 95% CI: 1.472-1.852) or mental health problem (AOR: 2.461, 95% CI: 1.998-3.030) were more likely to receive an appointment, whereas those without a health problem (AOR: 0.578, 95% CI: 0.497-0.673) were less likely. Patients who received services from GAP A were more likely to receive a medical appointment (AOR: 1.428, 95% CI: 1.311-1.555).

## Discussion

Building on a unique clinical-administrative dataset, this paper examined the socio-demographic, economic, and clinical characteristics of unattached adult patients and one GAP characteristic associated with receiving a medical appointment through the GAP. Results revealed that younger patients, those calling for acute health problems, medication renewals, or to have an administrative health form filled and those with physical or mental health problems were significantly more likely to receive medical appointments through the GAP. Additionally, patients from GAP A (vs. B) had higher odds of receiving a medical appointment. These findings are valuable to better understand the characteristics of individuals who use GAP services to improve the appropriate use of scarce healthcare resources in Quebec.

### Differences related to patient age

Patients aged 18-65 were more likely to receive a medical appointment than older adults (≥66 years). Although health does not change linearly and equally in older adults, this result seems counter-intuitive given that physical health problems, chronic illnesses, and cognitive impairments requiring medical follow-up are more prevalent in this population.^30-33^ In fact, older adults in our study were more likely to have a chronic disorder and be considered vulnerable (priority code A, B, or C). A possible explanation is that the GAP was mainly designed to address acute health problems, and more complex needs requiring follow-up care (multiple chronic disorders, polypharmacy, home care, etc.) might not fit well in this model. These hypotheses are supported by data (not shown) indicating that older adults’ requests more likely resulted in orientation to GAP transitional professional services for unstable chronic illnesses and home care support. The interaction between age and main reason for calling was also significant. In fact, younger adults were more likely to receive a medical appointment than older adults with the same reason for calling. Although the GAP has improved access to primary care services in Quebec, it might not be suitable for older adults with chronic conditions requiring follow-up care and raises questions about lack of relational continuity. Therefore, efforts are needed to ensure the GAP and its complementary services (e.g., transitional professional services) respond to the needs of older unattached patients, which represented 30% of our study sample. Also, younger people might wait longer before calling the GAP, leading to worse health problems that more likely necessitate a medical appointment. Work schedules can leave little room for calling the GAP, which can take several hours before being assessed by a nurse. Conversely, older adults might have more time to call or be curious about the service’s mission or functioning. After the study period, in fall 2023, a digital GAP (https://gap.soinsvirtuels.gouv.qc.ca/en)—an on-line platform where patients can fill a request regarding their health need—was implemented province-wide to reduce phone wait times and standardize GAP processes and trajectories. This solution will likely increase access to GAP services for all age groups and occupations.

### Variable medical capacity across GAPs

Patients calling GAP A were more likely to receive a medical appointment than patients calling GAP B. One possible explanation is differences in medical appointment availability for GAP patients. In fact, the characteristics of the local health territories showed that no medical appointment was available in 0.8% of cases in GAP A compared to 11.4% in GAP B. Within the GAP A territory, a medical clinic opened a site with a large appointment capacity especially for GAP patients. This might have led to patients that could have been seen by another health professional being granted a medical appointment, suggesting that available resources influence the orientation of GAP patients. In the GAP context, the responsibility for caring for unattached patients and the allocation of appointments to these patients has always relied on the voluntary participation of clinics and family physicians, which creates territorial inequalities in access. A recent provincial report highlighted territorial differences in terms of available medical appointments for GAP patients impacting the capacity of GAPs to respond to local needs.^34^ Indeed, in our study, 6% of GAP A patients who needed an appointment did not receive one, compared to 14% in GAP B.

### Main reasons for receiving a medical appointment

Our findings showed that acute health problems, medication renewals, and filling an administrative health form were associated with a greater likelihood of receiving a medical appointment. Similarly, Bergeron et al. showed that acute health problems and medication renewals were determinants of needing a medical appointment in paediatric patients.^25^ In Quebec, the scope of practice of community pharmacists was recently extended to increase flexibility and autonomy to initiate and modify medications and renew prescriptions.^35^ This new legislation seems particularly beneficial for unattached patients given that community pharmacists are easy to access.^36^ As part of the GAP, efforts were made in the region under study to collaborate with community pharmacists and orient patients to these services when appropriate.^37^ Thus, our finding regarding the increased likelihood of receiving a medical appointment for medication renewals may be surprising. However, community pharmacists cannot renew prescriptions beyond 12 months.^35^ Further, community pharmacists’ scope of practice was expanded around the same time as the GAP and involved important practice changes that could take time to implement. Sustained efforts are needed to support and collaborate with community pharmacists to maximize the use of their expertise and increase their role in patient care.

In Quebec, many administrative health forms, including from the Quebec Automobile Insurance Society, the Quebec Labour Standards Commission, private insurers, and employers, must be signed by a physician. Indeed, patients needing a form filled were ten times more likely to receive a medical appointment. This is worrying given the physician shortage and their increased administrative workload, which limits time for patient care. To tackle this issue, Quebec’s Ministry of Health recently started revising health administrative forms needing to be filled by physicians.^38^

### Strengths and limitations

This study is the first to document the characteristics of adult GAP patients and the determinants of receiving a medical appointment through the GAP. Data were extracted retrospectively from EMR, and the reliability of administrative data for research purposes remains a challenge. Although the study was conducted in only one region, it included a large population of patients who called the GAP during the study period.

Given that data were not collected for research and information collected varied during the study period, multiple information was considered to determine if patients received an appointment. For ease of analysis, only one call per patient was considered. When identifying the call to include, we prioritized that which resulted in a medical appointment given that for the same health problem, a call can lead to a medical appointment up to several weeks later. This might have overestimated the associations given that appointments were more likely received during retained than excluded calls. However, this potential bias might be limited given that 67% of the sample called only once during the study period. As part of this study, we were not able to capture patient outcomes related to GAP service received such as patient satisfaction and ER visits. However, in the next part of our larger study, we will assess, through a self-reported survey, the patient experience associated with GAP services received including unmet healthcare needs, ER consultations and healthcare services that patients would have used if the GAP did not exist. This will inform whether GAP service trajectories (medical appointment, referral to community pharmacist and other professionals, orientation to emergency department) are meeting patients’ needs.

## Conclusion

This is the first study to document the characteristics of patients using the GAP, an organizational innovation aiming to facilitate unattached patients’ navigation and access to primary care in Quebec. The findings can inform on the characteristics of patients with potential needs not being meet by the GAP in order to improve care trajectories. In addition to providing a socio-demographic portrait of patients receiving medical appointments through the GAP, the findings highlighted the reason of consultations for which unattached patients received medical appointments. Together, this information could support the development and improvement of care trajectories (e.g., filling administrative forms and medication renewal) and thus, make better use of scarce human and financial resources in primary healthcare systems. Future studies on patient-reported experience measures are needed to better understand if GAP services are meeting unattached patient needs.
